# Supplementary material for: Artificial intelligence enables precision diagnosis of cervical cytology grades and cervical cancer
Source: Nat Commun. 2024 May 22;15:4369. doi: 10.1038/s41467-024-48705-3 (PMC11111770; doi:10.1038/s41467-024-48705-3)
Supplement: Supplementary file 1 — Supplementary information [file 41467_2024_48705_MOESM1_ESM.pdf]

Supplementary information

Supplementary information to: Artificial Intelligence Enables Precision Diagnosis of Cervical Cytology Grades and Cervical Cancer

**Supplementary Figure 1**	**Study design of the training and validation datasets.**
**Supplementary Figure 2**	**Study design of the randomised observational trial.**
**Supplementary Figure 3**	**Comparisons of sensitivity, specificity and accuracy of the AICCS alone, cytopathologists, and AICCS-assisted cytopathologists in the randomised observational trial.**
**Supplementary Figure 4**	**The interface of the AICCS system.**
**Supplementary Figure 5**	**The AICCS system assists the diagnosis of cervical cytology grades.**
**Supplementary Figure 6**	**An example of quality control.**
**Supplementary Figure 7**	**Heat maps to visualize the outputs of the patch-level detection model.**
**Supplementary Figure 8**	**Procedure of classification on the patch-level and WSI-level.**
**Supplementary Figure 9**	**The top 20 features used in the WSI classification model.**
**Supplementary Table 1**	**Average precision in the patch-level detection and classification.**
**Supplementary Table 2**	**Performance of four deep learning algorithms in cervical cytopathological diagnosis.**
**Supplementary Table 3**	**Abbreviations and definitions.**
**Supplementary Table 4**	**Distribution of cervical cytology grades in the training and validation datasets.**
**Supplementary Table 5**	**Distribution of cervical cytology grades in the randomised observational trial.**

Supplementary Table 6 **Performance of the AICCS in the validation datasets.**

Supplementary Table 7 **Negative predictive value of the AICCS in the validation datasets.**

Supplementary Table 8 **Negative predictive value of the AICCS alone, cytopathologists, and AICCS-assisted cytopathologists in the prospective validation datasets and randomised observational trial.**

Supplementary Table 9 **Performance of the AICCS alone or cytopathologists in cervical cytopathological diagnosis with reference to histopathological diagnosis in the SYSMH internal validation dataset.**

Supplementary Table 10 **Abbreviation list and morphology of each classification.**

Figure legends

Supplementary Figure 1. Study design of the training and validation datasets.

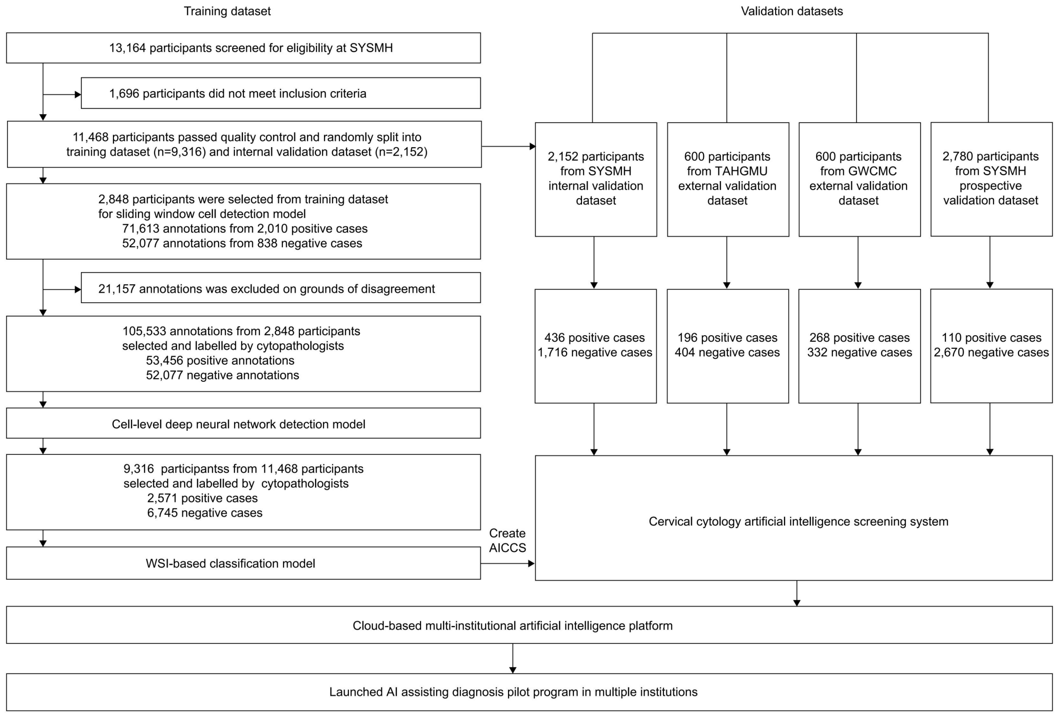

During the training phase, we retrospectively obtained cervical cytology images from 11,468 eligible individuals at SYSMH. These images were randomly split at a 4:1 ratio into a training cohort (n = 9,316) and an internal validation cohort (n = 2,152) used for training and evaluating the AICCS, respectively. Moving to the validation phase, we initially validated the AICCS using the SYSMH internal validation dataset (n = 2,152), followed by the GWCMC (n = 600) and TAHGMU (n = 600) external validation datasets. We prospectively collected cervical cytology images from an additional 2,780 eligible participants at SYSMH to further assess the AICCS's generalizability and robustness in clinical practice. AICCS, Artificial Intelligence Cervical Cancer Screening System. SYSMH, Sun Yat-sen Memorial Hospital. GWCMC, Guangzhou Women and Children’s Medical Center. TAHGMU, The Third Affiliated Hospital of Guangzhou Medical University.

Supplementary Figure 2. Study design of the randomised observational trial.

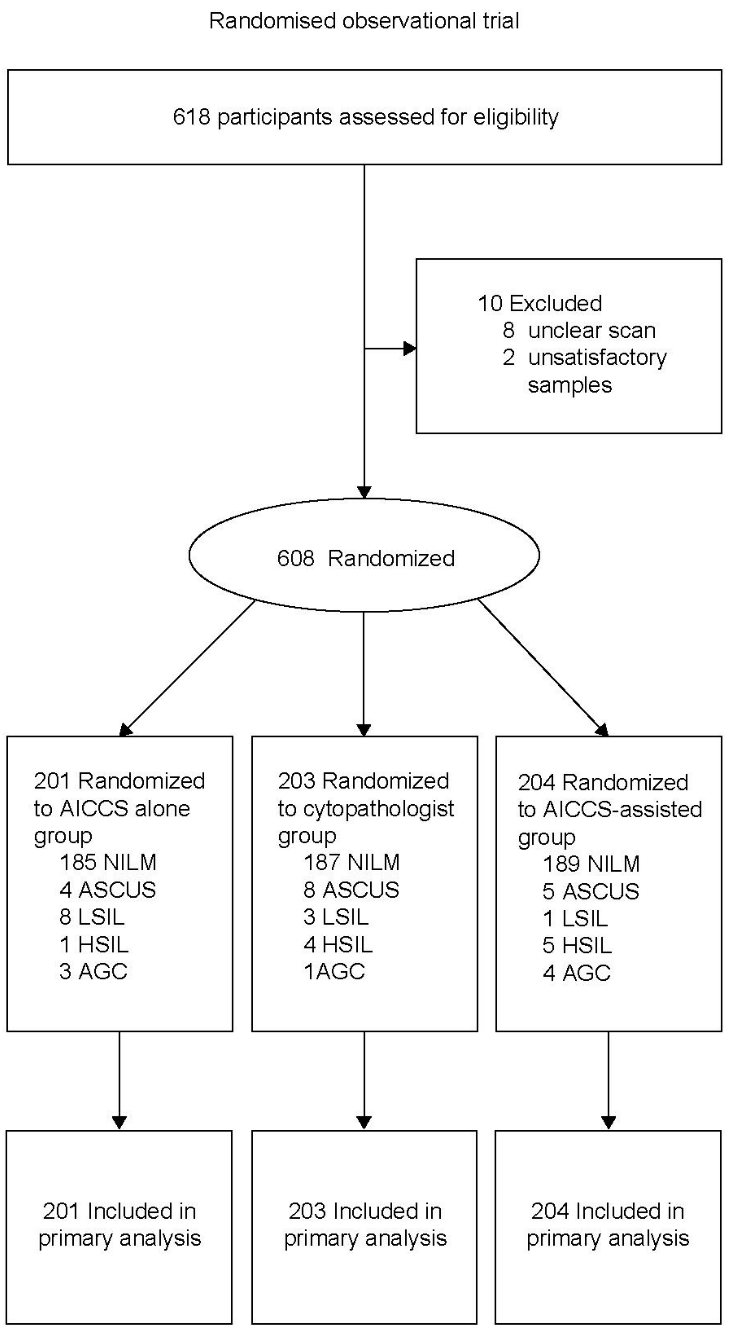

We randomly assigned 608 participants who passed quality control into three groups (1:1:1 ratio) in the randomised observational trial stage. The first group received diagnosis solely from the AICCS alone (n = 201), the second group received diagnosis from cytopathologists (n = 203), and the third group received diagnosis from cytopathologists assisted by the AICCS (n = 204). NILM, Negative for intraepithelial lesion or malignancy. ASC-US, Atypical squamous cells of undetermined significance. LSIL, Low-grade squamous intraepithelial lesions. HSIL, High-grade squamous intraepithelial lesions. AGC, Atypical glandular cells.

Supplementary Figure 3. Comparisons of sensitivity, specificity and accuracy of the AICCS alone, cytopathologists, and AICCS-assisted cytopathologists in the randomised observational trial.

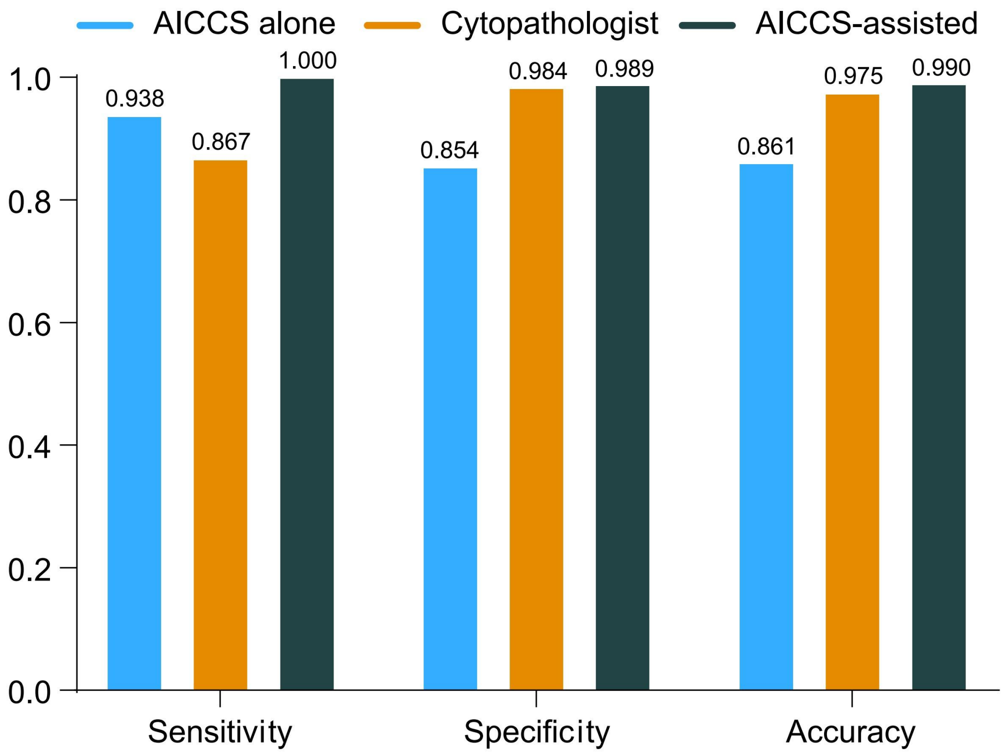

Source data are provided as a Source Data file.

Supplementary Figure 4. The interface of the AICCS system.

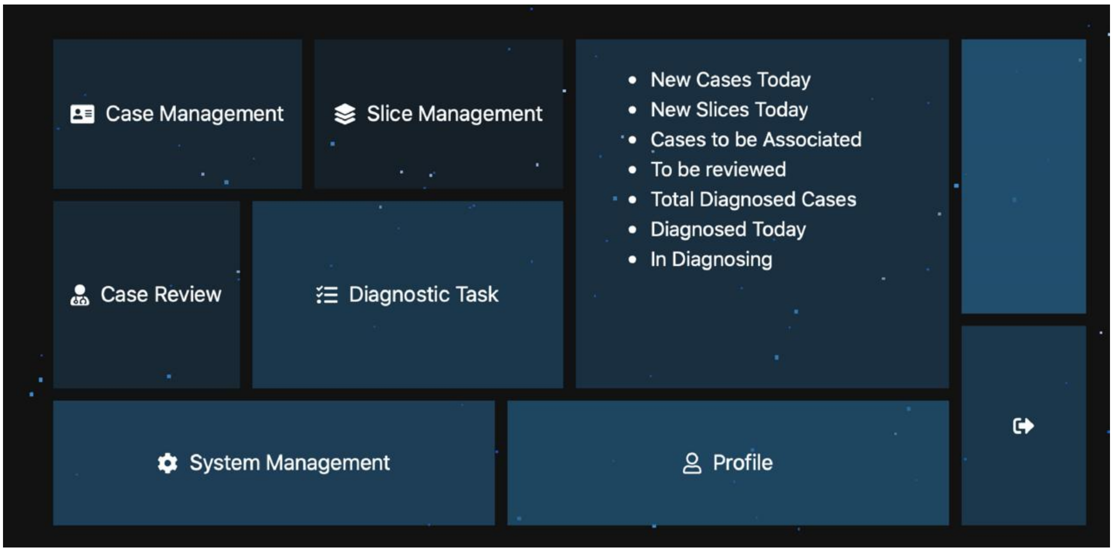

A website was set up to make the AICCS publicly available(https://ai-eng.cellsvision.com:3443/).

Supplementary Figure 5. The AICCS system assists the diagnosis of cervical cytology grades.

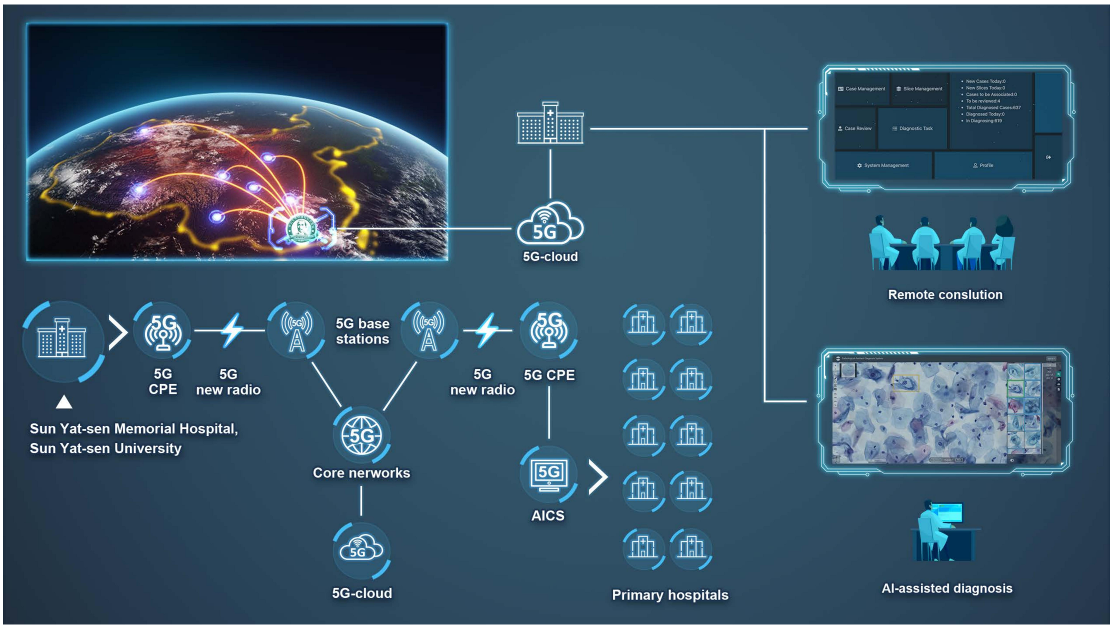

For patients coming to a collaborating hospitals for a thin-prep cytologic test, the system is able to provide two key clinical applications as follows: 1) the precision diagnosis of cervical cytology grades can be obtained with the assistance of the AICCS, with the cervical cytology smears uploaded to the AICCS to increase the accuracy of diagnosis; and 2) the AICCS provides free access as a consulting service for patients and clinicians after their have uploaded their WSI on the AICCS. Experienced experts can discuss complex cases and reach consensus in diagnosis. AICCS, Artificial Intelligence Cervical Cancer Screening System; 5G, Fifth generation.

Supplementary Figure 6. An example of quality control.

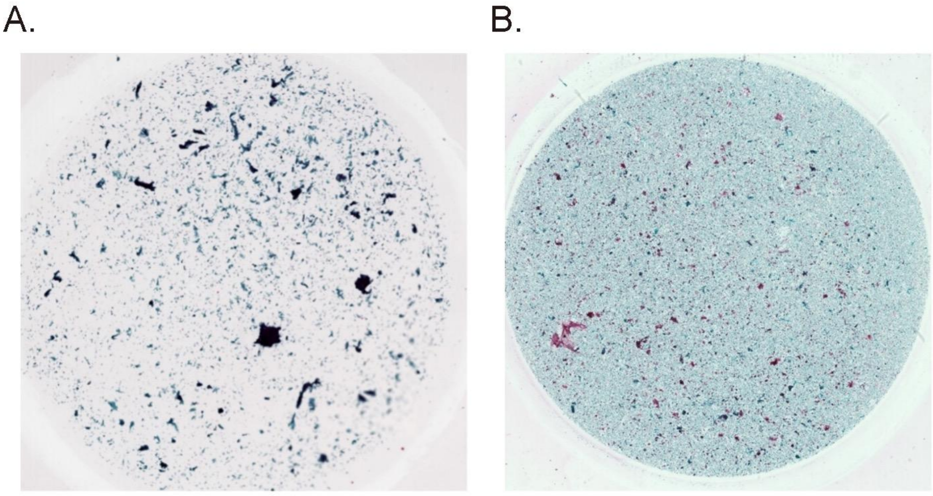

Quality control measures were instituted by conducting a thorough assessment of participant eligibility and adhering to stringent criteria for specimen selection. Within the framework of the AICCS, an AI-assisted methodology was employed to identify and address potential issues related to scanning quality throughout the digitization process. To support this objective, an image classification model was developed. This model leverages thumbnail images of WSIs to detect occurrences of scanning quality impediments, including but not limited to blurriness (A) and instances of incomplete scanning. Lastly, the WSIs pass quality control move on to AICCS system (B).

Supplementary Figure 7. Heat maps to visualize the outputs of the patch-level detection model.

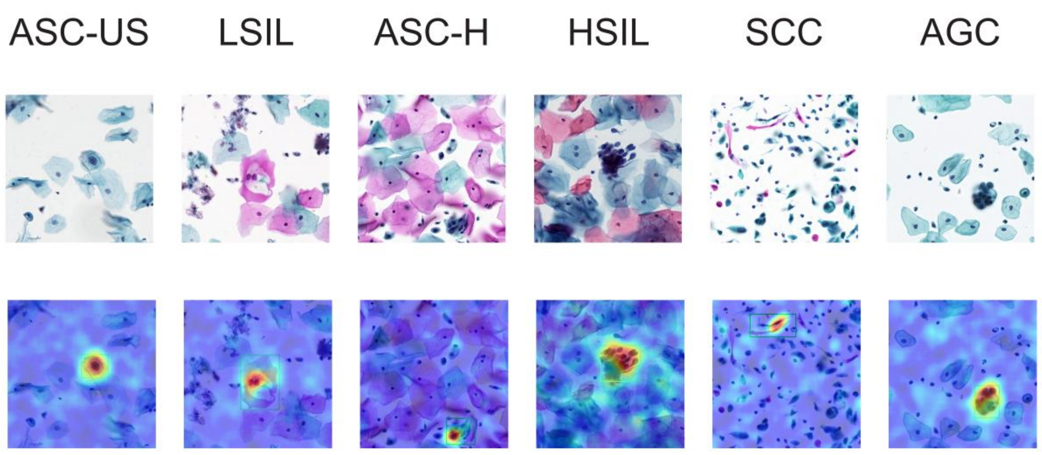

ASC-US, Atypical squamous cells of undetermined significance. LSIL, Low-grade squamous intraepithelial lesions. ASC-H, Atypical squamous cells - cannot exclude HSIL. HSIL, High-grade squamous intraepithelial lesions. SCC, Squamous cell carcinoma. AGC; Atypical glandular cells.

Supplementary Figure 8. Procedure of classification on the patch level and WSI level.

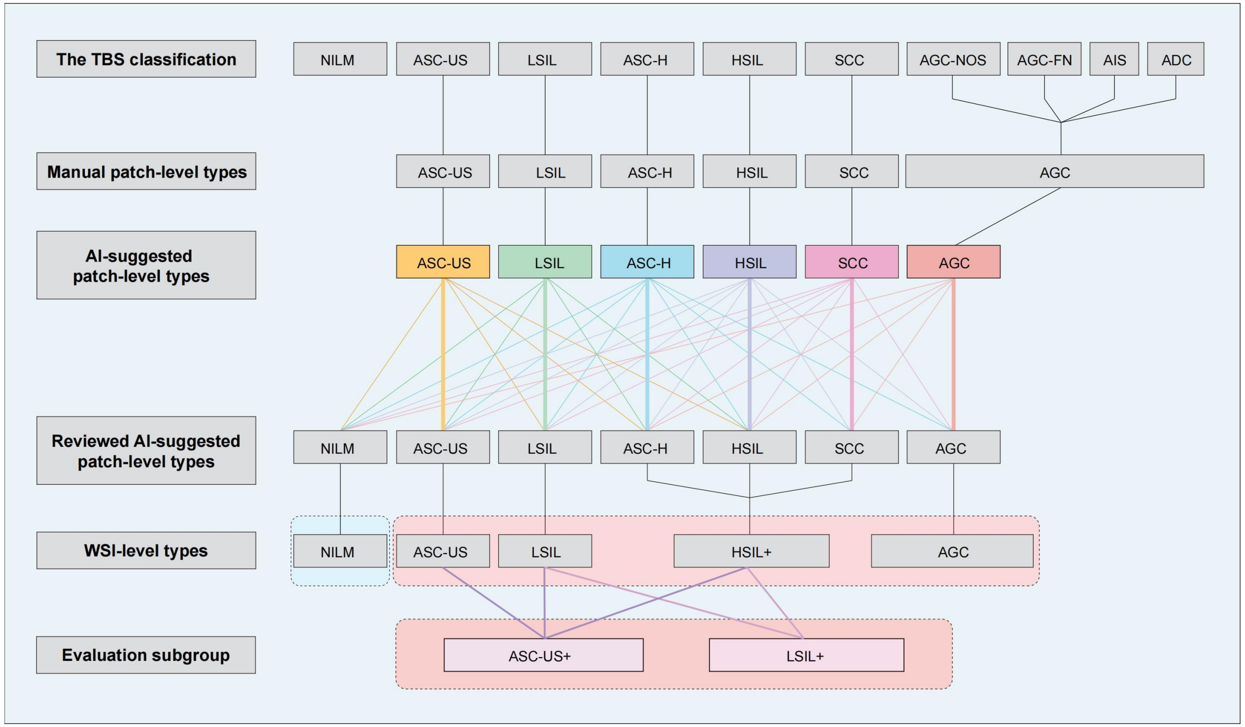

AI, Artificial Intelligence. TBS, The Bethesda System. SYSMH, Sun Yat-sen Memorial Hospital. GWCMC, Guangzhou Women and Children Medical Center. TAHGMU, The Third Affiliated Hospital of Guangzhou Medical University. AUC, Area under the receiver operating characteristic curve. NILM, Negative for intraepithelial lesion or malignancy. ASC-US, Atypical squamous cells of undetermined significance. LSIL, Low-grade squamous intraepithelial lesions. ASC-H, Atypical squamous cells - cannot exclude HSIL. HSIL, High-grade squamous intraepithelial lesions. SCC, Squamous cell carcinoma. AGC, Atypical glandular cells. AGC-NOS, Atypical glandular cells, not otherwise specified. AGC-FN, Atypical glandular cells, favor neoplastic. AIS, Endocervical adenocarcinoma in situ. ADC, Adenocarcinoma. ASC-US+ includes ASC-US, LSIL, ASC-H, HSIL, and SCC. LSIL+ includes LSIL ASC-H, HSIL, and SCC. HSIL+ includes ASC-H, HSIL and SCC.

Supplementary Figure 9. The top 20 features used in the WSI classification model.

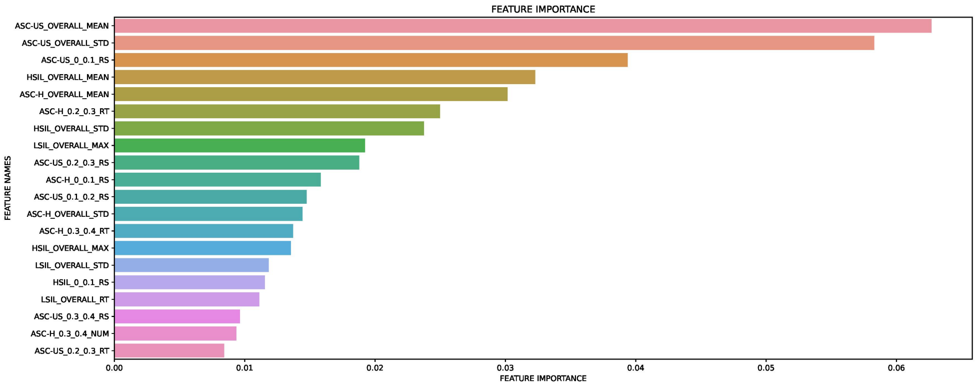

The feature names consist of detected cell name, detected confidence range, and statistic metric. Detected cell name is one of patch-level category, including ASC-US, LSIL, ASC-H, HSIL, SCC, or AGC. Detected confidence range is between 0.0 and 1.0, with interval of 0.1. Statistic metric refers to the number of cells (NUM) as follows: RS, Ratio of cells in this range to total cells for the same category. RT, Ratio of cells in this range to total detection of all categories. MAX, The maximum of confidence scores for objects in each category. MEAN, Average of confidence scores for objects in each category. STD, Standard deviation of confidence scores for objects in each category.

Supplementary Tables

Supplementary Table 1 Average precision in the patch-level detection and classification.

	**Retina**			**Faster-R-CNN**		
	AP_{10}	AP_{30}	AP_{50}	AP_{10}	AP_{30}	AP_{50}
All classes	50.90	47.31	34.68	51.93	47.29	34.65

AP_{10}, Average precision for the patch-level detection results at IOU of 10%. AP_{30}, Average precision for the patch-level detection results at IOU of 30%. AP_{50}, Average precision for the patch-level detection results at IOU of 50%. Source data are provided as a Source Data file.

Supplementary Table 2. Performance of four deep learning algorithms in cervical cytopathological diagnosis

	Cell detection			WSI classification			Sensitivity	Specificity	Accuracy	NPV	PPV	AUC
	Network	Backbone	Hyperparameters	Features	Classifier	Hyperparameters	(95% CI)	(95% CI)	(95% CI)	(95% CI)	(95% CI)	(95% CI)
Model 1	Faster-R-CNN	ResNet50	Patch size: 1,024×1,024 Lr: 0.0001–0.00001 Optimizer: sgd Momentu: 0.90	Cell distributions	DNN	Layer: 383×512×512×5, Lr: 0.0001, Optimizer: sgd, Momentu:0.90, Drop rate: 0	0.711 (0.665–0.754)	0.797 (0.777–0.816)	0.780 (0.762–0.798)	0.919 (0.904–0.932)	0.460 (0.420–0.499)	0.830 (0.805–0.855)
Model 2	Faster-R-CNN	ResNet50	Patch size: 1,024×1,024 Lr: 0.0001–0.00001 Optimizer: sgd Momentu: 0.90	Cell distributions	Random forest	Cv:5 Class_weight: {0: 0·3, 1: 1, 2: 0·8, 3: 0·9, 4: 2}, criterion: entropy, Max_depth: 30, Max_features: 80 N_estimators: 300	0.836 (0.796–0.871)	0.889 (0.873–0.903)	0.879 (0.864–0.892)	0.959 (0.948–0.968)	0.636 (0.593–0.677)	0.918 (0.899–0.937)
Model 3	Retina	ResNet18	Patch size: 1,024×1,024 Lr: 0.01–0.002 Optimizer: sgd Momentu: 0.90	Cell distributions	DNN	Layer: 383×256×256×5, Lr: 0.0001 Optimizer: sgd, Momentu:0.90 Drop rate: 0.20	0.538 (0.490–0.585)	0.945 (0.933–0.955)	0.862 (0.847–0.876)	0.889 (0.874–0.903)	0.712 (0.660–0.760)	0.830 (0.805–0.854)
Model 4	Retina	ResNet18	Patch size: 1,024×1,024 Lr: 0.01–0.002 Optimizer: sgd Momentu: 0.90	Cell distributions	Random forest	Cv: 4 Class_weight: {0: 0·2, 1: 0·5, 2: 1, 3: 1, 4: 0·8}, criterion: entropy max_depth: 10, max_features: 50 n_estimators: 200	0.906 (0.875–0.932)	0.874 (0.857–0.889)	0.881 (0.866–0.894)	0.980 (0.971–0.987)	0.647 (0.608–0.685)	0.922 (0.904–0.940)

A total of 9,316 images from SYSMH were assigned into the training dataset, and 2,152 images from SYSMH were assigned into the internal validation dataset for algorithms' development and evaluation. WSI, Whole-slide Image. RCNN, Region Convolutional Neural Networks. DNN, Deep Neural Networks. NPV, Negative predictive values. PPV, Positive predictive values. AUC, Area under the curve. Lr, Learning rate. Cv, Cross validation. Sgd, Stochastic gradient descent. Source data are provided as a Source Data file.

Supplementary Table 3. Abbreviations and definitions.

Abbreviation	Definition
ADC	Adenocarcinoma
AGC	Atypical glandular cells
AGC-FN	Atypical glandular cells, favor neoplastic
AGC-NOS	Atypical glandular cells, not otherwise specified
AICCS	Artificial Intelligence Cervical Cancer Screening
AIS	Endocervical adenocarcinoma in situ
ASC-H	Atypical squamous cells - cannot exclude a HSIL
ASC-US	Atypical squamous cells of undetermined significance
AUC	Area under the curve
CNN	Convolutional neural network
CI	Confidence interval
DNN	Deep neural network
FPN	Feature pyramid network
GWCMC	Guangzhou Women and Children Medical Center
HPV	Human papillomavirus
HDFF	Hybrid deep feature fusion
HSIL	High-grade squamous intraepithelial lesions
LSIL	Low-grade squamous intraepithelial lesions
MAX	Maximum
NILM	Negative for intraepithelial lesion or malignancy
NMPA	The National Medical Products Administration
NPV	Negative predictive value
NUM	Number
PPV	Positive predictive values
ROI	Region of interest
R-CNN	Region-based convolutional neural network
RGB	Red green blue
RS	Ratio of cells in this range to total cells for the same category
RT	Ratio of cells in this range to total detection of all categories
SaaS	Software-as-a-Service
SCC	Squamous cell carcinoma
SDA	Stain density absorbance
SSD	Single-shot detector
STD	Standard deviation
SYSMH	Sun Yat-sen Memorial Hospital
TAHGMU	The Third Affiliated Hospital of Guangzhou Medical University
TBS	The Bethesda System
WSI	Whole-slide image
YOLO	You only look once
5G	Fifth generation

Supplementary Table 4. Distribution of cervical cytology grades in the training and validation datasets

Cervical cytology grade	SYSMH training dataset (N= 9,316)	SYSMH internal validation dataset (N= 2,152)	GWCMC external validation dataset (N= 600)	TAHGMU external validation dataset (N= 600)	SYSMH prospective validation dataset (N= 2,780)
NILM (%)	6,745 (72.4)	1,716 (79.7)	332 (55.3)	404 (67.3)	2,670 (96.0)
ASC-US (%)	730 (7.8)	94 (4.4)	13 (2.2)	38 (6.3)	53 (1.9)
LISL (%)	995 (10.7)	172 (8.0)	90 (15.0)	91 (15.2)	25 (0.9)
ASC-H (%)	279 (3.0)	50 (2.0)	41 (6.8)	24 (4.0)	9 (0.3)
HSIL (%)	401 (4.3)	92(4.6)	85 (14.2)	34(5.7)	17(0.6)
SCC (%)	35 (0.4)	5 (0.2)	4 (0.7)	0 (0)	2 (0.1)
AGC (%)	131 (1.4)	23 (1.1)	35 (5.8)	9 (1.5)	4 (0.1)

SYSMH, Sun Yat-sen Memorial Hospital; GWCMC, Guangzhou Women and Children Medical Center. TAHGMU, The Third Affiliated Hospital of Guangzhou Medical University. NILM, Negative for intraepithelial lesion or malignancy. ASC-US, Atypical squamous cells of undetermined significance. LSIL, Low-grade squamous intraepithelial lesions. ASC-H, Atypical squamous cells - cannot exclude HSIL. HSIL, High-grade squamous intraepithelial lesions. SCC, Squamous cell carcinoma. AGC; Atypical glandular cells. Source data are provided as a Source Data file.

Supplementary Table 5. Distribution of cervical cytology grades in the randomised observational trial

Cervical cytology grade	AICCS alone (N= 203)	cytopathologists (N= 201)	AICCS-assisted cytopathologists (N= 204)
NILM (%)	187 (92.1)	185 (92.0)	184 (90.2)
ASC-US (%)	8 (3.9)	4 (2.0)	5 (2.5)
LISL (%)	3 (1.5)	8 (4.0)	1 (0.5)
HSIL (%)	4 (2.0)	1 (0.5)	5 (2.5)
AGC (%)	1 (0.5)	3 (1.5)	4 (2.0)

AICCS, Artificial Intelligence Cervical Cancer Screening System. NILM, Negative for intraepithelial lesion or malignancy. ASC-US, Atypical squamous cells of undetermined significance. LSIL, Low-grade squamous intraepithelial lesions. HSIL, High-grade squamous intraepithelial lesions. AGC, Atypical glandular cells. Source data are provided as a Source Data file.

Supplementary Table 6 Performance of the AICCS in the validation datasets

	SYSMH internal validation dataset (N= 2,152)	GWCMC external validation dataset (N= 600)	TAHGMU external validation dataset (N= 600)
All cervical cytology grades			
Sensitivity (95% CI)	0.906 (0.875–0.932)	0.902 (0.859–0.935)	0.918 (0.868–0.953)
Specificity (95% CI)	0.874 (0.857–0.889)	0.810 (0.763–0.850)	0.811 (0.770–0.847)
Accuracy (95% CI)	0.881 (0.866–0.894)	0.850 (0.819–0.878)	0.843 (0.812–0.872)
AUC (95% CI)	0.922 (0.904–0.939)	0.909 (0.883–0.934)	0.875 (0.840–0.909)
Stratification analysis			
NILM			
Sensitivity (95% CI)	0.874 (0.857–0.889)	0.810 (0.763–0.850)	0.811 (0.770–0.847)
Specificity (95% CI)	0.911 (0.879–0.936)	0.935 (0.895–0.963)	0.925 (0.876–0.960)
Accuracy (95% CI)	0.881 (0.867–0.895)	0.861 (0.829–0.888)	0.844 (0.813–0.873)
AUC (95% CI)	0.923 (0.912–0.934)	0.929 (0.908–0.950)	0.879 (0.852–0.905)
ASC-US+			
Sensitivity (95% CI)	0.911 (0.879–0.936)	0.935 (0.895–0.963)	0.925 (0.876–0.960)
Specificity (95% CI)	0.874 (0.857–0.889)	0.810 (0.763–0.850)	0.811 (0.770–0.847)
Accuracy (95% CI)	0.881 (0.867–0.895)	0.861 (0.829–0.888)	0.844 (0.813–0.873)
AUC (95% CI)	0.923 (0.905–0.941)	0.929 (0.905–0.953)	0.879 (0.844–0.913)
LSIL+			
Sensitivity (95% CI)	0.928 (0.894–0.954)	0.962 (0.927–0.984)	0.955 (0.904–0.983)
Specificity (95% CI)	0.874 (0.858–0.889)	0.810 (0.763–0.850)	0.811 (0.770–0.847)
Accuracy (95% CI)	0.883 (0.868–0.896)	0.869 (0.837–0.896)	0.845 (0.812–0.874)
AUC (95% CI)	0.950 (0.933–0.967)	0.946 (0.924–0.968)	0.927 (0.896–0.959)
HSIL+			
Sensitivity (95% CI)	0.980 (0.942–0.996)	0.944 (0.889–0.977)	0.889 (0.708–0.977)
Specificity (95% CI)	0.874 (0.857–0.889)	0.810(0.763–0.850)	0.811 (0.770–0.847)
Accuracy (95% CI)	0.882 (0.867–0.897)	0.846 (0.810–0.878)	0.815 (0.776–0.850)
AUC (95% CI)	0.960 (0.938–0.982)	0.930 (0.899–0.962)	0.896 (0.816–0.975)

All cervical cytology grades include NILM, ASC-US, LSIL, ASC-H, HSIL, SCC and AGC. ASC-US+ includes ASC-US, LSIL, ASC-H, HSIL and SCC. LSIL+ includes LSIL, ASC-H, HSIL, and SCC. HSIL+ includes ASC-H, HSIL and SCC. AICCS, Artificial Intelligence Cervical Cancer Screening. SYSMH, Sun Yat-sen Memorial Hospital. GWCMC, Guangzhou Women and Children Medical Center. TAHGMU, The Third Affiliated Hospital of Guangzhou Medical University. AUC, Area under the receiver operating characteristic curve. NILM, Negative for intraepithelial lesion or malignancy. ASC-US, Atypical squamous cells of undetermined significance. LSIL, Low-grade squamous intraepithelial lesions. ASC-H, Atypical squamous cells - cannot exclude HSIL. HSIL, High-grade squamous intraepithelial lesions. SCC, Squamous cell carcinoma. AGC, Atypical glandular cells. Source data are provided as a Source Data file.

Supplementary Table 7. Negative predictive value of the AICCS in the validation datasets.

	SYSMH internal validation dataset (N= 2,152)	GWCMC external validation dataset (N= 600)	TAHGMU external validation dataset (N= 600)
NPV	0.973 (0.964, 0.981)	0.913 (0.875, 0.942)	0.958 (0.931, 0.976)

AICCS, Artificial Intelligence Cervical Cancer Screening. NPV, Negative predictive value. SYSMH, Sun Yat-sen Memorial Hospital. GWCMC, Guangzhou Women and Children Medical Center. TAHGMU, The Third Affiliated Hospital of Guangzhou Medical University. Source data are provided as a Source Data file.

Supplementary Table 8. Negative predictive value of the AICCS alone, cytopathologists, and AICCS-assisted cytopathologists in the prospective validation datasets and randomised observational trial.

	AICCS alone	Cytopathologists	AICCS-assisted cytopathologists
Prospective validation datasets	0.997 (0.995, 0.999)	0.996 (0.993, 0.998)	1.000 (0.998, 1.000)
Randomised observational trial	0.994 (0.965, 1.000)	0.989 (0.9962, 0.999)	1.000 (0.980, 1.000)

AICCS, Artificial Intelligence Cervical Cancer Screening. Source data are provided as a Source Data file.

Supplementary Table 9. Performance of the AICCS alone or cytopathologists in cervical cytopathological diagnosis with reference to histopathological diagnosis in the SYSMH internal validation dataset

	AICCS alone	Cytopathologists	AICCS alone vs Cytopathologists
			P value
All cervical cytology grades			
Sensitivity (95% CI)	0.965 (0.920–0.989)	1.000 (0.974–1.000)	0.101
Accuracy (95% CI)	0.836 (0.771–0.889)	0.861 (0.798–0.910)	0.540
Precision (95% CI)	0.862 (0.798–0.911)	0.861 (0.798–0.910)	0.979
Stratification analysis			
LSIL+			
Sensitivity (95% CI)	0.970 (0.925–0.992)	1.000 (0.972–1.000)	0.136
Accuracy (95% CI)	0.833 (0.765–0.888)	0.868 (0.804–0.918)	0.641
Precision (95% CI)	0.854 (0.788–0.906)	0.868 (0.804–0.918)	0.966
HSIL+			
Sensitivity (95% CI)	1.000 (0.938–1.000)	1.000 (0.937–1.000)	NA
Accuracy (95% CI)	0.728 (0.618–0.821)	0.740 (0.628–0.834)	0.861
Precision (95% CI)	0.725 (0.614–0.819)	0.740 (0.626–0.834)	0.899

A total of 165 individuals with cytopathological diagnoses and histopathological diagnoses from the SYSMH internal validation dataset were selected to conduct a diagnosis analysis. All cervical cytology grades included NILM, ASC-US, LSIL, ASC-H, HSIL, SCC and AGC. LSIL+ includes LSIL, ASC-H, HSIL, and SCC. HSIL+ includes ASC-H, HSIL and SCC. AICCS, Artificial Intelligence Cervical Cancer Screening System. SYSMH, Sun Yat-sen Memorial Hospital. LSIL, Low-grade squamous intraepithelial lesions. HSIL, High-grade squamous intraepithelial lesions. SCC, Squamous cell carcinoma. The $\chi 2$ test (two-sided) was used for two-group categorical variables. Statistical significance was considered when the two-tailed P-value was less than 0.05. Source data are provided as a Source Data file.

Supplementary Table 10. Abbreviation list and morphology of each classification.

Abbreviations	The TBS classification	Corresponding patch-level types	Corresponding WSI-level types	Definition
NILM	Negative for intraepithelial lesion or malignancy	NILM	NILM	Specimens with no epithelial abnormalities
ASC-US	Atypical squamous cells-undetermined significance	ASC-US	ASC-US	Squamous epithelial cells with enlarged nucleus and slight nuclear membrane irregularity, which refers to changes that are suggestive of LSIL
LSIL	Low-grade squamous intraepithelial lesion	LSIL	LSIL	Nuclear enlargement more than three times the area of normal intermediate nuclei, or the cells with diagnostic koilocytic features have a sharply defined perinuclear cavity
ASC-H	Atypical squamous cells- cannot exclude a HSIL	ASC-H	HSIL+	A designation reserved for the minority of atypical squamous cells (ASC) cases (expected to represent less than 10 % of all ASC interpretations) in which the cytologic changes are suggestive of HSIL
HSIL	High- grade squamous intraepithelial lesion	HSIL	HSIL+	The cells are smaller and show less cytoplasmic maturity than cells of LSIL, nuclei are generally hyperchromatic and coarsely granular, and contour of the nuclear membrane is quite irregular
SCC	Squamous cell carcinoma	SCC	HSIL+	An invasive epithelial tumor composed of squamous cells of varying degrees of differentiation, some of which show the nuclear features of HSIL
AGC-NOS	Atypical glandular cells, not otherwise specified	AGC	AGC	Atypical glandular cells (AGC) include atypical endocervical and endometrial cell lesions, which including AGC-NOS, AGC-FN, AIS, and ADC
AGC-FN	Atypical glandular cells, favor neoplastic			
AIS	Endocervical adenocarcinoma in situ			
ADC	Adenocarcinoma			

TBS, The Bethesda System.
