# Supplementary material for: Artificial intelligence enables precision diagnosis of cervical cytology grades and cervical cancer
Source: Nat Commun. 2024 May 22;15:4369. doi: 10.1038/s41467-024-48705-3 (PMC11111770; doi:10.1038/s41467-024-48705-3)
Supplement: Supplementary file 3 — Reporting Summary [file 41467_2024_48705_MOESM3_ESM.pdf]

nature portfolio

Corresponding author(s): Herui Yao, Nengtai Ouyang, and Jin Wang.

Last updated by author(s): 21/3/24

Reporting Summary

Nature Portfolio wishes to improve the reproducibility of the work that we publish. This form provides structure for consistency and transparency in reporting. For further information on Nature Portfolio policies, see our Editorial Policies and the Editorial Policy Checklist.

Statistics

For all statistical analyses, confirm that the following items are present in the figure legend, table legend, main text, or Methods section.

n/a	Confirmed	
☐	☒	The exact sample size (*n*) for each experimental group/condition, given as a discrete number and unit of measurement
☒	☐	A statement on whether measurements were taken from distinct samples or whether the same sample was measured repeatedly
☐	☒	The statistical test(s) used AND whether they are one- or two-sided<br>*Only common tests should be described solely by name; describe more complex techniques in the Methods section.*
☒	☐	A description of all covariates tested
☒	☐	A description of any assumptions or corrections, such as tests of normality and adjustment for multiple comparisons
☐	☒	A full description of the statistical parameters including central tendency (e.g. means) or other basic estimates (e.g. regression coefficient) AND variation (e.g. standard deviation) or associated estimates of uncertainty (e.g. confidence intervals)
☐	☒	For null hypothesis testing, the test statistic (e.g. *F*, *t*, *r*) with confidence intervals, effect sizes, degrees of freedom and *P* value noted<br>*Give P values as exact values whenever suitable.*
☒	☐	For Bayesian analysis, information on the choice of priors and Markov chain Monte Carlo settings
☒	☐	For hierarchical and complex designs, identification of the appropriate level for tests and full reporting of outcomes
☒	☐	Estimates of effect sizes (e.g. Cohen's *d*, Pearson's *r*), indicating how they were calculated

Our web collection on statistics for biologists contains articles on many of the points above.

Software and code

Policy information about availability of computer code

Data collection	There is no commercial, open source and custom code used to collect the data in this study.
Data analysis	Python (version 3.6) and Medcala (version 15) are using for data analysis.

For manuscripts utilizing custom algorithms or software that are central to the research but not yet described in published literature, software must be made available to editors and reviewers. We strongly encourage code deposition in a community repository (e.g. GitHub). See the Nature Portfolio guidelines for submitting code & software for further information.

Data

Policy information about availability of data

All manuscripts must include a data availability statement. This statement should provide the following information, where applicable:

- Accession codes, unique identifiers, or web links for publicly available datasets
- A description of any restrictions on data availability
- For clinical datasets or third party data, please ensure that the statement adheres to our policy

The datasets are governed by data usage policies specified by the data controller (Sun Yat-sen Memorial Hospital, Sun Yat-sen University). The WSIs, codes and expected output involved in the main text are securely maintained by the Ethics Committee of Sun Yat-sen Memorial Hospital. Access to these resources for non-commercial research endeavors is contingent upon approval by the corresponding author (yaoherui@mail.sysu.edu.cn) through a request deemed reasonable and

sanctioned by the Ethics Committee.
In this study, the digital scanner (PRECICE 600 (UNIC TECHNOLOGIES, INC.), KF-PRO-400-HI (Ningbo Jiangfeng Bio-Information Technology Co., Ltd.)) specific data reading SDK packages under commercial license were used for WSI importing. The networks used in our AICCS system were developed in Python (version 3.6). Our patch level abnormal cell detection was based on RetinaNet(https://github.com/jkznst/RetinaNet-mxnet, an unofficial implementation of ICCV 2017 RetinaNet (Focal Loss)). The WSI level classification algorithm was based on Random Forest (scikit-learn 0.23.2). The related codes are stored in https://github.com/cellsvision/AICCS.

Research involving human participants, their data, or biological material

Policy information about studies with human participants or human data. See also policy information about sex, gender (identity/presentation), and sexual orientation and race, ethnicity and racism.

Reporting on sex and gender	The study is aimed to develop a precision diagnosis of cervical cytology grades and cervical cancer, therefore all participants included in this study are female.
Reporting on race, ethnicity, or other socially relevant groupings	Owing to the study implementation centers for this study being located in China, the majority of the participants included in this research are Chinese, with the ethnicity being predominantly of the Asian subgroup.
Population characteristics	The population are 18 years or older, not pregnant, and didn't have undergone cervical resection with primary malignancies.
Recruitment	Participants, 18 years or older, not pregnant, without mental illness or cognitive impairment, and who consented to cervical liquid-based cytology for definite diagnosis, were included. Exclusion criteria were participants who had undergone cervical resection with primary malignancies.
Ethics oversight	The study protocol with ID: 2020-KY-114 was approved by Ethics committee, Sun Yat-Sen Memorial Hospital of Sun Yat-Sen University.

Note that full information on the approval of the study protocol must also be provided in the manuscript.

Field-specific reporting

Please select the one below that is the best fit for your research. If you are not sure, read the appropriate sections before making your selection.

☒ Life sciences ☐ Behavioural & social sciences ☐ Ecological, evolutionary & environmental sciences

For a reference copy of the document with all sections, see nature.com/documents/nr-reporting-summary-flat.pdf

Life sciences study design

All studies must disclose on these points even when the disclosure is negative.

Sample size	Sample size calculation for randomized controlled trial: The calculation was performed in the tests for paired AUC module of PASS software (version 15.0.5), which needed to set the power, significance level, AUC of test1, and AUC of test2. The significance level (0.025) was controlled and split between the analyses of AICCS alone group vs cytopathologists group (0.02) and AICCS-assisted cytopathologists group vs cytopathologists (0.005).<br>For AICCS vs. cytopathologists: The AUC of cytopathologists was 0.85 versus the expected AUC of AICCS was 0.91. Using a one-sided 0.02 significance level and 80% power it is calculated that 196 subjects per group are required for the study.<br>For AICCS-assisted cytopathologists vs. AICCS: The expected AUC of AICCS was 0.91 versus the expected AUC of AICCS-assisted cytopathologists was 0.97, using a one-sided 0.005 significance level and 80% power it is calculated that 175 subjects per group are required for the study.<br>Taken together, allowing for a 20% drop-out rate, more than a total of 588 patients will need to be randomized.
Data exclusions	Between January 2016 and December 2020, a total of 16,056 eligible participants were enrolled in this multicenter study. Distinct datasets were created, including retrospective and prospective population-based datasets, as well as a randomized controlled trial. In the retrospective dataset, 13,1641 participants were screened for eligibility at SYSMH, and 1,696 participants did not meet the inclusion criteria for the reason that samples with fewer than 5,000 visible cells, uncovered squamous epithelial cells, or those affected by blood, inflammatory cells, epithelial cell overlapping, poor fixation, excessive drying, or unknown component contamination affecting over 75% of squamous epithelial cells,which were excluded from the analyses. No participants were excluded in GWCMC and TAHGMU from retrospective dataset.<br>No participants were excluded on prospective population-based datasets.<br>The randomized controlled trial was conducted at SYSMH from August 13, 2020, to December 14, 2020. A total of 618 participants were screened for eligibility, among which eight participants were excluded for unclear scan, and two participants were excluded for unsatisfactory samples. Finally, 608 participants who met our inclusion criteria were further analyses.
Replication	This is a clinical study, and experiments were neither replicated or performed independently. But the results of this study can be reproduced.
Randomization	Randomization was conducted in the randomize controlled trial. The participants who passed quality control were randomized (1:1:1) to receive a diagnosis from cytopathologists, AICS, or with AICS assistance. Randomization was done via a random number generation program with no stratification factors to avoid selection bias. The computerized randomization sequence was performed blindly by an independent statistician using a random assignment sequence. Study investigators assessing outcomes and analysing the data were not masked to diagnosis assignments.

Blinding	The investigators were blinded to group allocation during data collection and/or analysis.

Reporting for specific materials, systems and methods

We require information from authors about some types of materials, experimental systems and methods used in many studies. Here, indicate whether each material, system or method listed is relevant to your study. If you are not sure if a list item applies to your research, read the appropriate section before selecting a response.

Materials & experimental systems

n/a	Involved in the study	
☒	☐	Antibodies
☒	☐	Eukaryotic cell lines
☒	☐	Palaeontology and archaeology
☒	☐	Animals and other organisms
☐	☒	Clinical data
☒	☐	Dual use research of concern
☒	☐	Plants

Methods

n/a	Involved in the study	
☒	☐	ChIP-seq
☒	☐	Flow cytometry
☒	☐	MRI-based neuroimaging

Clinical data

Policy information about clinical studies

All manuscripts should comply with the ICMJE guidelines for publication of clinical research and a completed CONSORT checklist must be included with all submissions.

Clinical trial registration	NCT04551287
Study protocol	The study protocol is attached below the email.
Data collection	This study enrolled a total of 16,056 eligible participants was consisted of a multicenter study, a randomized controlled trial, and a prospective real-world dataset in China. We built several distinct datasets: retrospective and prospective population-based datasets, randomized controlled trial to train and validate the AICCS for the diagnosis of cervical cytology grade and cervical cancer through analysis of diverse cervical cytological whole slide images (WSIs) data.<br>In the multicenter study, eligible individuals' slides for the cervical cytology screening respectively collected from the Sun Yat-sen Memorial Hospital (SYSMH, Guangzhou, China) between January, 2016 and January, 2020, were randomly assigned to the training dataset and the SYSMH internal validation dataset in order to train the Artificial Intelligence Cervical Cancer Screening (AICCS) System. Then, eligible slides respectively collected from the other two participating hospitals between January 2016 and January 2020 were used as two independent external validation datasets (Guangzhou Women and Children Medical Center [GWCMC, Guangzhou, China] and The Third Affiliated Hospital of Guangzhou Medical University [TAHGMU, Guangzhou, China]).<br>Further, a prospective validation dataset was conducted to distinguish the diagnostic performance of the cytopathologists, AICCS, and AICCS-assisted cytopathologists, in which slides from eligible individuals were prospectively obtained between August 28, 2020 and October 16, 2020 at SYSMH.
Outcomes	The primary outcome is Area under ROC curve (AUC). The secondary outcome are sensitivity, specificity, accuracy.
